# Malaria Transmission Intensity and Parasitemia during the Three-Dose RTS,S/AS01 Vaccination Series do not Reduce Magnitude of Antibody Response nor Efficacy Against the First Case of Malaria

**DOI:** 10.21203/rs.3.rs-2960373/v1

**Published:** 2023-05-25

**Authors:** Griffin J Bell, Stephaney Gyaase, Varun Goel, Bright Adu, Benedicta Mensah, Paulin Essone, David Dosoo, Musah Osei, Karamoko Niare, Kenneth Wiru, Katerina Brandt, Michael Emch, Anita Ghansah, Kwaku Poku Asante, Tisungane Mvalo, Selidhi Todagbe Agnandji, Jonathan J Juliano, Jeffrey A Bailey

**Keywords:** vaccine, Africa, geographic information system, GIS, immunology, delayed malaria, rebound malaria

## Abstract

**Background:**

RTS,S/AS01 has been recommended by WHO for widespread implementation in medium to high malaria transmission settings. Previous analyses have noted lower vaccine efficacies in higher transmission settings, possibly due to the more rapid development of naturally acquired immunity in the control group.

**Methods:**

To investigate a reduced immune response to vaccination as a potential mechanism behind lower efficacy in high transmission areas, we examine initial vaccine antibody (anti-CSP IgG) response and vaccine efficacy against the first case of malaria to exclude the delayed malaria effect using data from three study areas (Kintampo, Ghana; Lilongwe, Malawi; Lambaréné, Gabon) from the 2009–2014 phase III trial (NCT00866619). Our key exposures are parasitemia during the vaccination series and malaria transmission intensity. We calculate vaccine efficacy (one minus hazard ratio) using a cox-proportional hazards model and allowing for the time-varying effect of RTS,S/AS01.

**Results:**

We find that antibody responses to the primary three-dose vaccination series were higher in Ghana than in Malawi and Gabon, but that neither antibody levels nor vaccine efficacy against the first case of malaria varied by transmission intensity or parasitemia during the primary vaccination series.

**Conclusions:**

We find that vaccine efficacy is unrelated to infections during vaccination. Contributing to a conflicting literature, our results suggest that vaccine efficacy is also unrelated to infections before vaccination, meaning that delayed malaria is likely the main reason for lower efficacy in high transmission settings, not reduced immune responses. This may be reassuring for implementation in high transmission settings, though further studies are needed.

## Background

Malaria remains a public health challenge, with 241 million cases in 2020 despite the increasing implementation of control measures such as artemisinin combination therapies, insecticide-treated bed nets, and indoor residual spraying.(1) RTS,S/AS01, a vaccine recently recommended for widespread implementation by the World Health Organization (WHO), is a newly available tool to reduce this burden. RTS,S, administered with the adjuvant AS01, is a pre-erythrocytic vaccine targeting the circumsporozoite protein (CSP) of *Plasmodium falciparum*. Overall efficacy of the vaccine in phase III trials was modest, resulting in a 36.3% reduction in the incidence of clinical malaria over a median of 4 years in children aged 5–17 months across 11 sites in sub-Saharan Africa.(2) Efficacy varied between study areas, with areas of higher malaria transmission having lower vaccine efficacy, but with more cases averted (2). Understanding the reasons for efficacy heterogeneity is critical for the implementation of the current vaccine and the development of the next generation of malaria vaccines.

Various theories attempt to explain the geospatial heterogeneity in RTS,S/AS01 vaccine efficacy. (3) “Delayed” malaria, which is caused by the development of immunity from natural infections over time in the comparison group, is one explanation.(4,5) Since efficacy is a function of the ratio of incidences in the RTS,S/AS01 and comparison groups, greater natural immunity in the comparison group reduces RTS,S/AS01 efficacy, without necessarily changing the protection RTS,S/AS01 provides to vaccinated individuals. In higher transmission areas, more frequent natural infections result in faster development of protection in the comparison group over time. Since the protection afforded by RTS,S/AS01 wanes over time, it can even fall below the protection afforded to the comparison group by previous natural infections during later periods of follow-up in high transmission areas (known as “rebound malaria”).(5,6)

However, lower efficacies in higher transmission settings have elicited hypotheses that children in endemic regions experience immune modulation mediated through infections before or during vaccination (three doses over three months) resulting in lower immune responses. Two analyses of phase 2 data and one of phase III data found support for this hypothesis.(7–9) Potential mechanisms can be drawn from results identifying that RTS,S/AS01 induced fewer functional antibodies in older children with greater malaria exposure and suggesting that malaria infection prior to vaccination could result in worse T helper cell responses to vaccination.(7,8,10) Further, *P. falciparum* can modulate the immune response upon infection, resulting in the depletion of T cells and altering the functional characteristics of B cells, potentially reducing the immune response to vaccination.(11–14) Infections with other pathogens (such as helminths) concurrently with vaccination can reduce the immune response and efficacy of other malaria vaccines,(15,16) so it’s possible that concurrent malaria infection would have a similar effect.(17) Conversely, natural malaria infections expose children to a wider diversity of non-vaccine strains and antigens(18) resulting in broader antibody breadth that has been associated with protection against malaria.(19,20) In support of this, studies have predicted and shown that malaria vaccines that add additional antigens outperform their fewer-antigen counterparts.(21,22)

Here, we evaluated the impact of malaria infections, before or during vaccination, on RTS,S/AS01 antibody response and efficacy. From three phase III trial sites in Gabon, Ghana, and Malawi, we obtained longitudinal malaria infection data and baseline and post-vaccination anti-circumsporozoite antibody levels. Further, we obtained parasitemia counts during the three-dose vaccine schedule. We used ecological data at the individual, household, and neighborhood levels to estimate the background malaria transmission intensity as a proxy for pre-vaccination infections.

It is vital that we understand the specific mechanisms through which malaria transmission intensity does, or does not, alter the impact of RTS,S/AS01. With this understanding, we can improve implementation and design interventions to supplement RTS,S/AS01 vaccination where necessary.

## Methods

### Study Population and Design

Study participants included in this analysis were children (5–17 months) enrolled in the 2009–2014 phase III trial of RTS,S/AS01 in Kintampo, Ghana; Lilongwe, Malawi; and Lambaréné, Gabon. The details of the parent study can be found elsewhere.(2) Briefly, this was a randomized, double-masked, controlled clinical trial, which was stratified by age group (children 5–17 months and infants 6–12 weeks) and evaluated the efficacy of three- and four-dose regimens of RTS,S/AS01. In the three-dose regimen, RTS,S/AS01 was administered monthly, while the four-dose regimen added a booster dose around 18 months after the third dose. The primary outcome in the phase III trial was clinical malaria, defined as blood film microscopy measuring >5000 parasites per microliter and a fever within the previous 24 hours. Malaria surveillance was passive, except for 14, 16, and 18 months after the booster.

### Delayed/Rebound Malaria

Delayed malaria was a cause of reduced vaccine efficacy in high-incidence areas in the phase III trial of RTS,S/AS01.(5,6) In this analysis, we include only the first case of clinical malaria, post-vaccination, to exclude the impacts of the development of natural immunity in the comparison group.

### Primary Exposures: Malaria Transmission Intensity and Parasitemia During the Vaccination Series

Background malaria transmission intensity was estimated using our previously described method (5) leveraging household location and ecologic modeling. Briefly, we partitioned the data for phase III trial infants (6–12 weeks) who received the control vaccine into training and test datasets to fit a random forest model to estimate the relationship between 28 ecological variables and malaria incidence. Using the model fit, we can predict the malaria incidence for individual children (5–17 months). Though the actual malaria incidence experienced by the children in the time period between birth and vaccination likely does not map perfectly to this prediction, we theorize that a high positive correlation between the two makes predicted transmission intensity a good proxy.

Parasitemia during the vaccination series was measured directly by microscopy during passive surveillance in between the first and third dose of RTS,S: any individual which had a blood film microscopy with visible parasites (>0 parasites per microliter) was considered to be infected.

### Anti-CSP Antibody Response

Anti-CSP antibody (IgG against the NANP repeat region) levels, which were associated with protection at the time of the trial, were previously measured using standardized enzyme-linked immunosorbent assays (ELISA) on the day of the first dose and one month after the third dose of RTS,S/AS01.(23) Antibody data were collected for the first ~200 enrolled children at each site. To determine whether malaria transmission intensity influenced the anti-CSP antibody response to the RTS,S/AS01 vaccine, we fit a linear regression model with the outcome being either the natural log or untransformed antibody levels (whichever enabled the residuals to be approximately normally distributed, evaluated with a Quantile-Quantile plot, at the pre- and post- vaccine series timepoints). The covariates were vaccination status (RTS,S/AS01 or control) and the predicted malaria transmission intensity, as well as an interaction term between the two covariates. We also adjusted for the child’s age at the third dose; this age was set to the median when estimating from regression outputs. In case of any other differentiating study area characteristics besides transmission intensity, we fit an additional model where we adjusted for study area and included an interaction term between study area and vaccine group. The functional form of the malaria transmission intensity covariate was assessed by comparing Akaike information criterion (AIC) values. To determine whether infections during vaccination influenced the anti-CSP antibody response, we fit a linear regression model with antibody levels as the outcome and parasitemia during the vaccination series (dichotomous: ever >0 parasites per microliter) as the main exposure. Covariates and functional forms were treated in the same manner as the model for malaria transmission intensity.

### Efficacy and Time to First Malaria Case

To calculate the efficacy of RTS,S/AS01 against the first case of malaria depending on malaria transmission intensity setting and parasitemia during the vaccination series, we fit a Cox proportional hazards (CPH) model. The time period for the detection of the first malaria case was bounded by the date of the third vaccine and the earliest date among the first case of malaria, loss to follow-up, receipt of the fourth dose of RTS,S/AS01, or study end. All non-malaria outcomes were considered to be censored. Main covariates included vaccination status (RTS,S/AS01 or control), predicted malaria transmission intensity, and whether parasitemia during the initial 3-dose vaccination series period was observed. Interaction terms between treatment and intensity as well as treatment and parasitemia were included. We used repeated measures (six-month intervals) to allow for the effect of time on vaccine protection: an interaction term between time and treatment was included and we accounted for the repeated measures in standard error calculations. We also adjusted for the child’s age at the third vaccination; this age was set to the median when estimating from model outputs. Efficacy was calculated as one minus the hazard ratio for vaccination. Functional forms of transmission intensity were assessed using AIC.

Finally, we fit an accelerated failure time (AFT) model to estimate the impact of vaccination on the time to the first case of malaria. Censoring was conducted in the same manner as in the CPH model and we assumed the outcome followed a log-logistic distribution. Our covariates in the AFT model were the same as the CPH model. The functional form of transmission intensity was assessed using AIC.

### Sensitivity Analysis

We conducted a sensitivity analysis detailed in the supplemental materials by replacing our predicted transmission intensity values with average values from the Malaria Atlas Project over 2009–2014.(24) We repeated the relevant analysis with these values, including describing the relationship between transmission intensity and post-vaccination antibody response, as well as the CPH model.

## Results

### Study Population

In total, 2,427 children were included in this analysis, 992 from Ghana, 771 from Malawi, and 664 from Gabon (Table 1). Children (5–17 months) were oldest at primary vaccine series completion in Ghana (median 461 days) and youngest in Gabon (385 days). The estimated malaria transmission intensity was highest in Ghana. Though median transmission intensities were low in Malawi and Gabon, Malawi had more variation with a proportion of higher values. In terms of parasitemia during the vaccination series, it occurred in 23.2% of children in Ghana compared to less than 5% in Malawi and Gabon. Of these infections, one was detected on the same day as the first dose, 185 were detected in between the first and second doses, 26 were detected on the same day as the second dose, and 120 were detected in between the second and third doses. GPS coordinates were missing in 134 (20.2%) of the children from Gabon and thus we were unable to estimate their individual exposure (malaria transmission intensity value). Age at vaccination was missing in 31 total children (1.3%).

### Anti-CSP Antibody Response

**Figure 1:**
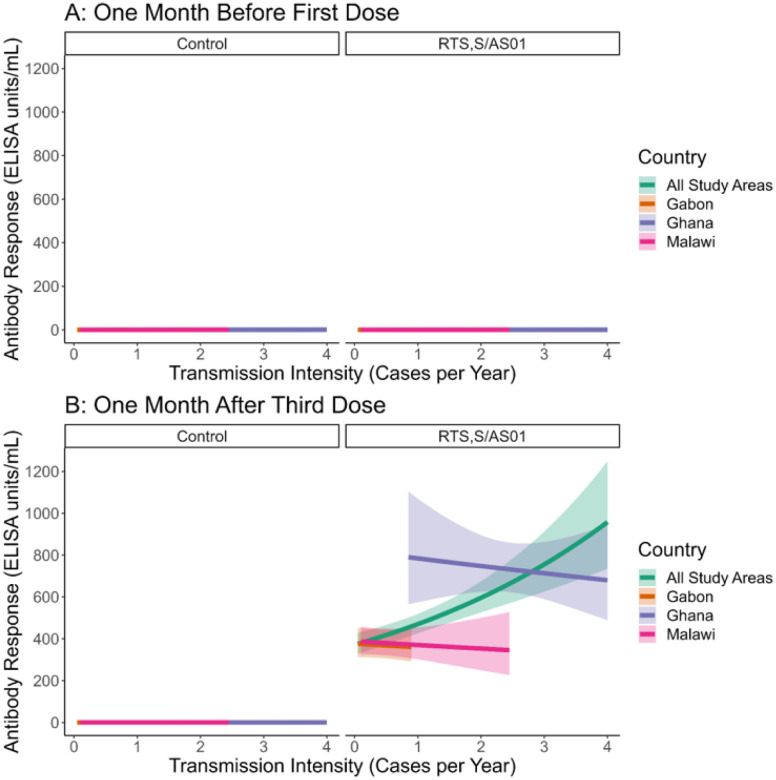
Anti-CSP Antibody Response by Transmission Intensity, All Study Areas and Study Area Adjusted (A: Pre-Vaccination, B: One Month Post-Vaccination) (A) Children have a very low anti-circumsporozoite protein (CSP) antibody response on the day of the first vaccine dose. (B) One month after vaccination, children in higher transmission areas have higher antibody responses to RTS,S/AS01 vaccination. This positive relationship disappears when adjusting for study area. Antibody responses were elevated in Ghana, the highest transmission study area.

Antibody data were available for 641 children (220 Ghana, 213 Malawi, 208 Gabon). On the day of vaccination with the first dose of RTS,S/AS01 or a control vaccine, participant anti-CSP levels were below 1.0 ELISA units/ml, regardless of malaria transmission intensity (Fig. 1A). One month after the third dose, those who received the control vaccine maintained low antibody levels, regardless of malaria transmission intensity, while those who received the three-dose RTS,S/AS01 vaccine series had greatly increased antibody levels (Fig. 1B). Among those who received RTS,S/AS01, antibody level was positively associated with transmission intensity: at 0.25 cases per person-year (CPPY), antibodies were estimated to be 393.8 ELISA units/ml and at 3 CPPY, antibodies were estimated to be at 755.8 ELISA units/ml. However, after study area stratification, we found no statistically significant relationship between transmission intensity and antibody response in the RTS,S/AS01 group (p = 0.82). Higher antibody responses and transmission intensities in Ghana were driving the positive correlation in the unstratified model (Fig. 1B, Supplemental Figs. 1 and 2). Controlling for transmission intensity, the antibody response to RTS,S/AS01 was higher in Ghana compared to the other sites. Older age at the third dose was negatively associated with antibody response before (p = 0.02) and after (p < 0.01) controlling for the study area. Our sensitivity analysis found a statistically significant positive relationship between malaria transmission intensity and 1-month post-vaccination antibody response (p = 0.02), which remained but became statistically insignificant after study area stratification (p = 0.48) (Supplemental Figs. 3 and 4).

**Figure 2:**
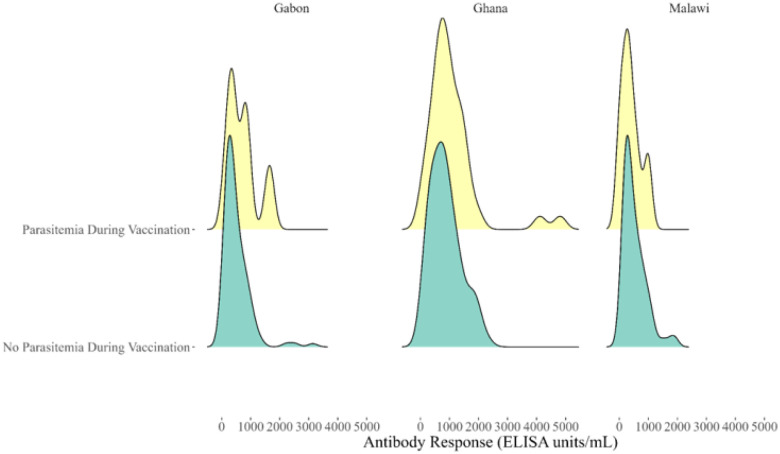
Anti-CSP Antibody Response (One Month Post-Vaccination) in RTS,S/AS01 Group

Parasitemia during the vaccination series did not increase antibody levels in the control group one month after the third dose: levels were near zero regardless of parasitemia. Those without parasitemia during RTS,S/AS01 vaccination developed lower antibodies levels, though this observed difference could have reasonably been generated by chance (p = 0.24). This difference remained statistically insignificant after stratification by study area (p = 0.68) (Fig. 2).

### Efficacy and Time to First Malaria Case

**Figure 3:**
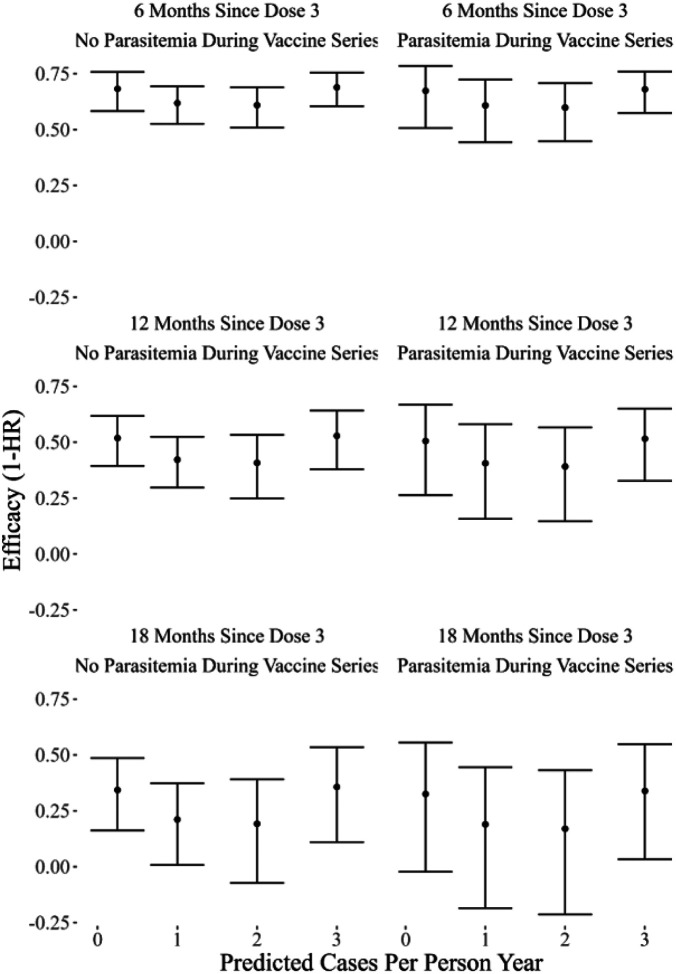
Efficacy Against the First Case of Malaria Over Time, Transmission Intensity, and Cases During Vaccination

In our CPH model, RTS,S/AS01 efficacy waned over time (Fig. 3). Evidence was weak that efficacy against the first case of malaria varied by transmission intensity or parasitemia during the three-dose vaccination series. Our sensitivity analysis found very similar results (Supplemental Fig. 5). Similar results were found using the AFT model, implying that the vaccine delayed the first case of malaria equally across transmission intensities, on the ratio scale (Supplemental Fig. 6). Age did not impact vaccine efficacy in a statistically significant manner (p = 0.47) when an interaction term was considered, thus, the interaction term was not included. A breakdown by site for the CPH model can be viewed in Supplemental Fig. 7, however, no differences were observed.

## Discussion

Following the phase III trial of RTS,S/AS01, multiple hypotheses were formed to explain the lower efficacy in higher malaria transmission areas.(3) One such hypothesis was that infections before and during RTS,S/AS01 vaccination reduce vaccine efficacy due to a lower immune response to vaccination. Based on transmission intensity modeling at the household level, our findings challenge this hypothesis. We found that both antibody response and efficacy against the first case of malaria were practically unrelated to transmission intensity and parasitemia during vaccination. Our sensitivity analysis found a statistically insignificant positive relationship between transmission intensity and antibody response, though it found no impact on vaccine efficacy against the first case of malaria. Antibody response was highest in Kintampo, Ghana, possibly for reasons other than transmission intensity, such as host differences.(25)

The existing literature is divided on whether infections before and during RTS,S/AS01 vaccination impact vaccine protection. One between-site study in Kintampo, Ghana and Manhiça, Mozambique found that post-vaccination antibody levels were positively associated with previous clinical malaria episodes and pre-vaccination antibody levels.(18) An analysis of all phase III trial sites found that higher baseline anti-CSP antibody levels were associated with higher anti-CSP antibody levels post-vaccination in children (5–17 months).(23) However, this effect was reversed in the infants (6–12 weeks), possibly related to the presence of maternal antibodies. Additionally, malaria exposure trains the immune system to hyper-respond to the stimulation of TLR2, potentially increasing anti-CSP antibody levels in higher transmission areas.(26) Higher anti-CSP antibody levels are correlated with increased RTS,S/AS01 vaccine efficacy in both high and low transmission settings.(23, 27) Other studies draw opposite conclusions. One between-site analysis found that RTS,S/AS01 efficacy was higher when malaria exposure before vaccination was lower, which the authors claimed was in line with the overall phase II and III trial findings that vaccine efficacy was lower in higher transmission sites.(2, 9, 28) However, the main trial result can be largely explained by “delayed malaria,” which reduces vaccine efficacy in higher transmission areas due to a faster development of naturally-acquired protection in the unvaccinated, not because of reduced protection from RTS,S/AS01.(4, 5) The authors also correctly acknowledge a major limitation in their study, that the greatest predictor of future malaria infection is past malaria infection.(9) When the authors controlled for confounding by transmission intensity (using study area as a proxy), associations between prior clinical malaria episodes and antibody response were reduced or lost.(9) Finally, a phase IIb study in Mozambique which used biomarkers as a proxy for malaria exposure found that complement-fixing antibodies were less readily induced in older (12–24 months) children with higher malaria exposure, though disentangling age and malaria exposure was difficult in this study.(7)

Our analysis has multiple strengths. First, evidence for the natural acquisition of immunity being delayed in the vaccine compared to the control group (resulting in delayed/rebound malaria) being largely responsible for lower efficacy in high transmission in areas is strong,(5, 6, 29, 30) and we intentionally filter out this effect by concentrating on antibody response to vaccination and the first malaria infection post-vaccination. Additionally, our vaccine efficacy calculations corroborate our vaccine antibody response results. Further, we used intrasite environmental data in order to model household-level malaria transmission intensity, improving upon studies that use study area as a proxy. This is especially important in sites with intrasite heterogeneity in transmission intensity. We also include a sensitivity analysis using more established transmission intensity estimates. This study also has limitations. First, we use transmission intensity as a proxy for infections before vaccination and this analysis would be improved if we had actual data on pre-vaccination infections. This is a problem throughout the existing literature: we could not find a study that directly measured pre-vaccination infections. Second, we rely on passive surveillance of malaria, so we may have missed some first cases or infections during vaccination, and our transmission intensity model (described elsewhere(5)) may underestimate transmission intensity in areas with lower healthcare access. Third, we consider here only the quantity of the anti-CSP antibody response. Previous research has shown that RTS,S vaccine efficacy is related to qualitative differences such as antibody isotypes.(31) Thus, focusing only on the size of the IgG anti-CSP NANP response may omit important correlates of protection against malaria.

## Conclusions

Our results suggest that the lower RTS,S/AS01 efficacy in higher malaria transmission areas may be largely due to delayed/rebound malaria, and not due to infections before or during vaccination which led to reduced immune responses. However, the literature remains divided over the influence of infections before vaccination on RTS,S/AS01 efficacy, with each published study (including ours) suffering from various important limitations. A future study that directly measures infections before vaccination is needed to elucidate the influence of such infections on RTS,S/AS01 efficacy. The role of rebound/delayed malaria, however, has a strong evidence base in the current literature. During the widespread rollout of RTS,S/AS01, it may be sensible to concentrate our energy on combating delayed malaria later in follow-up in order to maximize vaccine impact in high transmission areas.

## Figures and Tables

**Table 1:** Characteristics of the Phase III Trial Children (5–17 months)

Variable	Categorical: N (%), Numerical: Median (IQR)		
	*Ghana (N = 992)*	*Malawi (N=771)*	*Gabon (N = 664)*
Sex			
*Female*	483 (48.7%)	383 (49.7%)	307 (46.2%)
*Male*	509 (51.3%)	388 (50.3%)	357 (53.8%)
Age at Vaccination Series Completion (Days)			
*Median (IQR)*	461 (333.5, 539)	411.5 (316, 502.75)	385 (270.5, 495.5)
*Min - Max*	216–724	211–700	197–636
*Missing*	9	9	13
Vaccine Status			
*RTSS/AS01 (4 dose)*	332 (33.5%)	253 (32.8%)	218 (32.8%)
*RTS,S/AS01 (3 dose)*	330 (33.3%)	263 (34.1%)	223 (33.6%)
*Control*	330 (33.3%)	255 (33.1%)	223 (33.6%)
Estimated Malaria Transmission Intensity (Cases per Person-Year)			
*Median (IQR)*	2.30 (1.69, 2.81)	0.24 (0.18, 0.32)	0.30 (0.15, 0.50)
*Min - Max*	0.85–4.45	0.08–2.47	0.04–0.92
*Missing*	4	1	134
Parasitemia during the Vaccination Series			
*Yes*	230 (23.2%)	37 (4.8%)	26 (3.9%)
*No*	762 (76.8%)	734 (95.2%)	638 (96.1%)

## Data Availability

Phase III trial data are available upon reasonable request from GlaxoSmithKline (GSK), and ecological data used to build the malaria transmission intensity model are publicly available.
